# Tunable Dielectric Properties of Ferrite-Dielectric Based Metamaterial

**DOI:** 10.1371/journal.pone.0127331

**Published:** 2015-05-20

**Authors:** K. Bi, K. Huang, L. Y. Zeng, M. H. Zhou, Q. M. Wang, Y. G. Wang, M. Lei

**Affiliations:** State Key Laboratory of Information Photonics and Optical Communications & School of Science, Beijing University of Posts and Telecommunications, Beijing 100876, China; Northwestern Polytechnical University, CHINA

## Abstract

A ferrite-dielectric metamaterial composed of dielectric and ferrite cuboids has been investigated by experiments and simulations. By interacting with the electromagnetic wave, the Mie resonance can take place in the dielectric cuboids and the ferromagnetic precession will appear in the ferrite cuboids. The magnetic field distributions show the electric Mie resonance of the dielectric cuboids can be influenced by the ferromagnetic precession of ferrite cuboids when a certain magnetic field is applied. The effective permittivity of the metamaterial can be tuned by modifying the applied magnetic field. A good agreement between experimental and simulated results is demonstrated, which confirms that these metamaterials can be used for tunable microwave devices.

## Introduction

Dielectric materials play a very important role in the microwave communication systems because these materials are critical to realization of low-loss temperature-stable resonators and filters for satellite and broadcasting equipments, and many other microwave devices [1–3]. Most of dielectric materials for these devices show a low tunability and one of the easiest ways to improve the tunable property is to find materials where the parameters show magnetic DC bias dependence [4,5]. However, it is a great challenge for those materials to realize the tunable dielectric property in microwave and higher frequency bands [6,7]. The high tunability of their permittivity appears at low frequency region. Hence, dielectric materials with high tunability operating at higher frequency are urgently needed. The high frequency properties of current dielectric materials need to be further improved.

Metamaterials are a class of materials in which subwavelength features, rather than the constituent materials, control the macroscopic electromagnetic properties [8]. The unusual electromagnetic properties of metamaterials originate from the structure rather than being inherited directly from the materials, which opens a way to design material with more freedom [9–12]. Recently, Mie resonance-based metamaterials have been theoretical and experimentally studied [13–17]. Formed from dielectric resonators, dielectric metamaterial unit cells support an electric and magnetic dipole response due to Mie resonances [18]. Proper control of the lattice arrangement, resonator geometry, and composition allows control over the effective permittivity and permeability of the metamaterial [19–21]. In previous work, our group experimentally and numerically studied the magnetic Mie resonance of the metamaterial composed of dielectric cubes and ferrite cuboids, and obtained tunable effective permeability and permittivity in magnetic resonance mode [22]. However, the tunability of its permittivity is very low. Similar to the dielectric particles, the dielectric rods can also generate the Mie electromagnetic resonances. Peng *et al*. [23] demonstrated the second Mie resonance mode corresponds to the electric response, and consequently lead to negative permittivity. But, the resonance frequency of such Mie resonance metamaterials can not be tuned. Here, we report a high tunable dielectric property in the ferrite-dielectric metamaterial with electric resonance mode. The simulated and experimental results demonstrate the large effective permittivity of the metamaterial can be tuned by adjusting the applied magnetic field.

## Experimental

**Fig 1:**
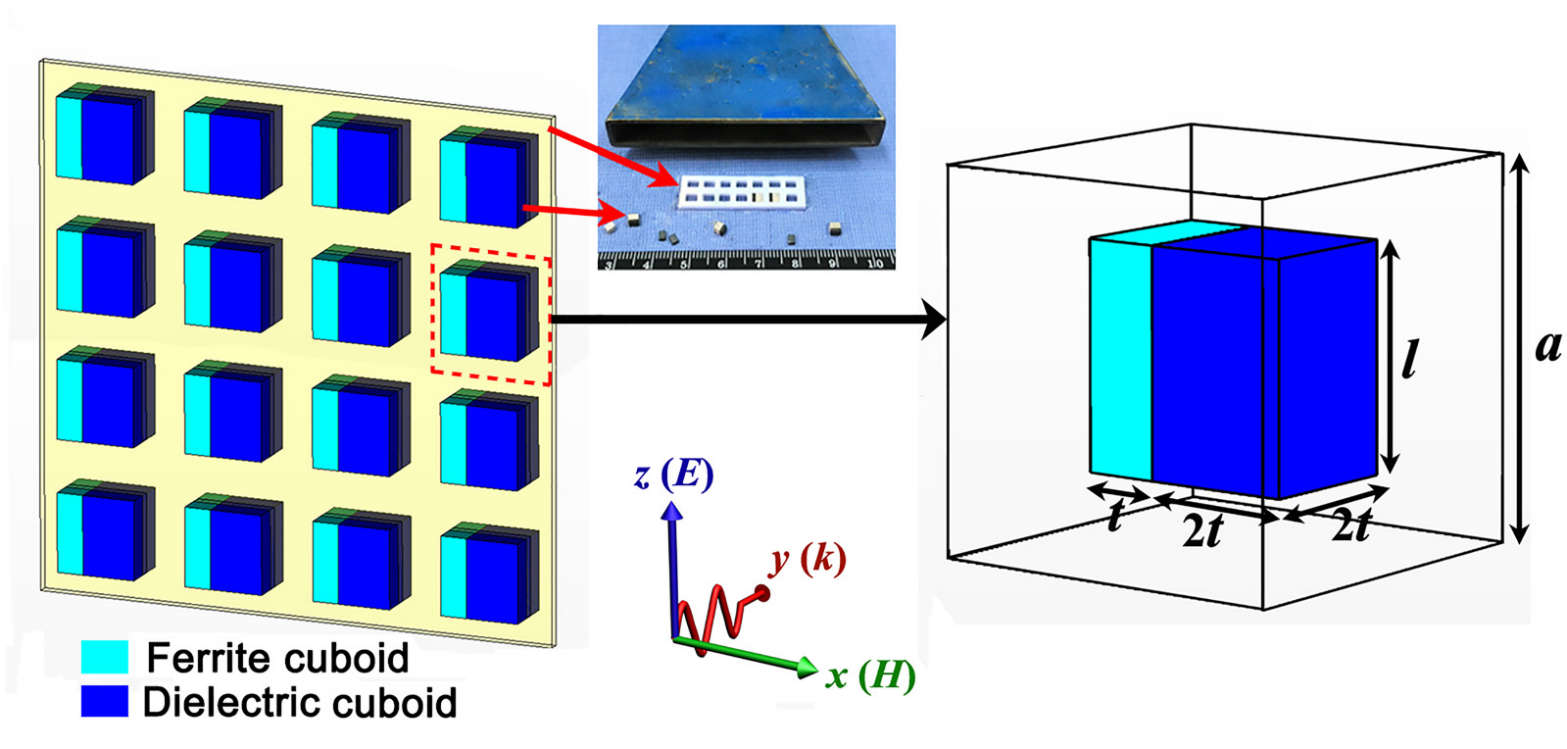
Schematic diagram of the ferrite-dielectric metamaterial.

The dielectric material chosen for this work was Barium Strontium Titanate (BST) ceramic. Employing the tape casting technique, green tapes were manufactured and sintered at 1400°C to produce BST slabs of thickness 2 mm. These slabs were then cut into cuboids of 2 × 2 × 3 mm^3^. The relative permittivity and dielectric loss are 206 and 0.015, which were measured by using dielectric rod resonator method [24]. The ferrite material chosen for this work was yttrium iron garnet (YIG) ferrite. YIG cuboids were cut to dimensions of 2 × 1 × 3 mm^3^. The saturation magnetization 4π*M*_s_, linewidth Δ*H*, and relative permittivity *ε*_r_ of the YIG cuboids were 1950 Gs, 10 Oe, and 14.5, respectively. The ferrite-dielectric metamaterial was fabricated by inserting the dielectric and ferrite cuboids into a Teflon substrate, as shown as an inset in Fig 1. The distance between the dielectric cubes in the *x* direction is the same as that in the *z* direction (5 mm). The inset shows a photograph of the metamaterial.

Numerical predictions of the transmission spectra were calculated by using the commercial time-domain package CST Microwave Studio TM. The dimension of the unit cell for the metamaterial was 5 × 5 × 5 mm^3^. All the parameters of the dielectric and ferrite cuboids were the same as those in the experiments. A plane wave propagates along *y* direction with the electric field along the *z* axis and the magnetic field along the *x* axis. The bias magnetic field was applied in the *z* direction. The microwave properties of the sample were measured by microwave measurement system, which is the same as that in Ref. [22].

## Results and Discussion

Dielectric cuboid is used to generate electric Mie resonance. The effective permittivity of the standard cylindrical dielectric resonator can be expressed by [25]

$$\varepsilon_{\text{eff}}\left(\text{a}\right)\approx \frac{\left(R^{2}-{\text{r}}^{2}\right)}{R^{2}}\left[1+I_{e}\left(r,R\right)\right],$$ (1)

$$I_{e}\left(r,R\right)=\frac{kr\varepsilon_{1}c_{0}^{\left(N\right)}J_{1}\left(k_{p}r\right)}{a_{0}^{\left(N\right)}\left[RH_{1}^{\left(1\right)}(kR)-rH_{1}^{\left(1\right)}(kr)\right]+RJ_{1}(kR)-rJ_{1}(kr)},$$ (2)

$$k=\omega {\left(\varepsilon_{0}\mu_{0}\right)}^{1/2},$$ (3)

$$k_{p}=\sqrt{\mu_{1}\varepsilon_{1}}k,$$ (4)

where the corresponding coefficients are defined in Ref. [25]. From Eqs (1–4), we observe that the effective permittivity *ε*_eff_ and permeability *μ*_eff_ can be not only influenced by the permittivity *ε*_2_ and the permeability *μ*_2_ of the dielectric cylinder, but also affected by the permittivity *ε*_1_ and the permeability *μ*_1_ of the background matrix. The unit cell of the metamaterial is composed of one dielectric cuboid and one ferrite cuboid. By interacting with the magnetic field of an electromagnetic wave, the ferromagnetic resonance can take place in the ferrite cuboid with applied magnetic field. The equation of the effective permeability for the ferrite under an applied magnetic field can be expressed by [26]

$$\mu_{1}=1-\frac{F\omega_{\text{mp}}^{2}}{\omega^{2}-\omega_{\text{mp}}^{2}-i\Gamma \left(\omega \right)\omega },$$ (5)

where Γ(*ω*) = [*ω* ^2^/(*ω*_r_+*ω*_m_)+*ω*_r_+*ω*_m_]*α*, *ω*_mp_ = (*ω*_r_(*ω*_r_+*ω*_m_))^1/2^, *ω*_m_ = 4π*M*_s_ *γ*, *ω*_r_ = *γH*, *α* is damping coefficient of ferromagnetic precession, *γ* is the gyromagnetic ratio, *F* = *ω*_m_/*ω*_r_, *ω*_m_ and *ω*_r_ are characteristic frequency and ferromagnetic resonance frequency of the ferrite, *M*_s_ is the saturation magnetization caused by the applied magnetic field, *H* is the applied magnetic field. According to Eq (5), the permeability of the ferrite can be tuned by adjusting the applied magnetic field. Because ferrite is one of the two important parts in this metamaterial, the permittivity of the metamaterial can be affected by the ferromagnetic resonance which can be tuned by applied magnetic field. To simplify fabrication, cuboids rather than cylinders were used as the dielectric materials to prepare the metamaterial.

**Fig 2:**
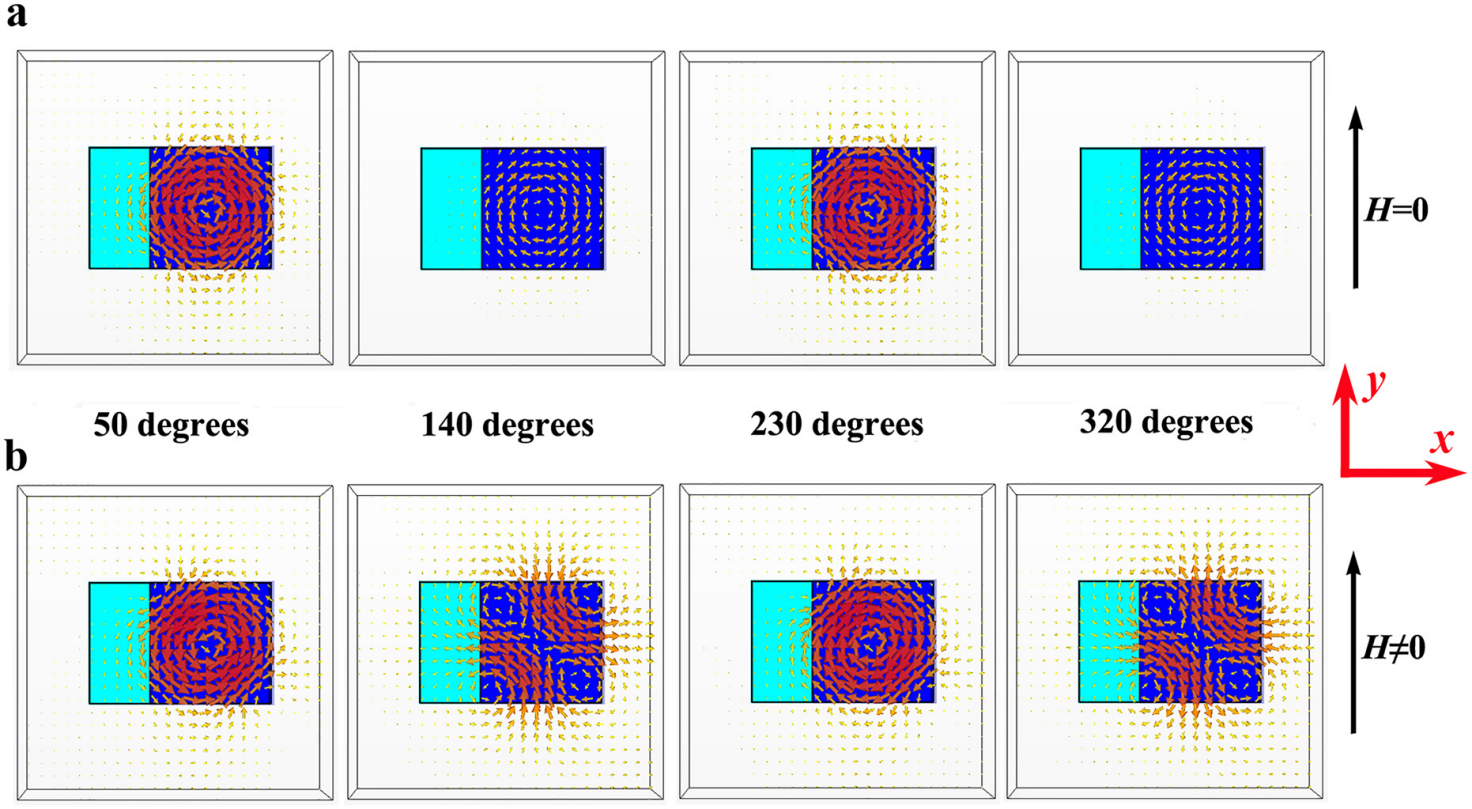
Magnetic field distributions at Mie resonance frequency for the unit cell (a) without and (b) with applied magnetic field, respectively. Four maps with different phase (50, 140, 230 and 320 degrees) are chosen to indicate the dynamic changes of magnetic field distributions in the unit cell.

To clarify the underlying physics of the applied magnetic field dependence of the electromagnetic properties of the metamaterial, the dynamic magnetic field distributions of the unit cell were simulated. Fig 2 shows the magnetic field distributions for the unit cell without or with applied magnetic field. Four maps with different phase (50, 140, 230 and 320 degrees) are chosen to show the dynamic changes of magnetic field distributions. When *H* = 0, the ferrite cuboid is not magnetized, and the dielectric cuboid plays a key role in interacting with electromagnetic wave. At the Mie resonance frequency of 11.07 GHz, as shown in Fig 2a, an induced circulation of displacement currents appears in the dielectric cuboid (*xy*-plane), which leads to a nonzero electric dipole momentum, resulting in a large electric field along *z* axis, demonstrating an electric resonance characteristic. The magnetic field distributions at 11.03 GHz for the unit cell with *H* > 0 are shown in Fig 2b. It can be seen that the magnetic field distributions at 50 and 230 degrees are much different from that shown in Fig 2a. This is because the ferromagnetic precession takes place in the ferrite cuboid, which could influence the Mie resonance of the dielectric cuboid. On the basis of the analysis of the magnetic field distributions for the unit cell without or with applied magnetic field, one can see that the electric Mie resonance can be influenced by the applied magnetic field. Hence, the applied magnetic field can affect the dielectric properties of the metamaterial.

**Fig 3:**
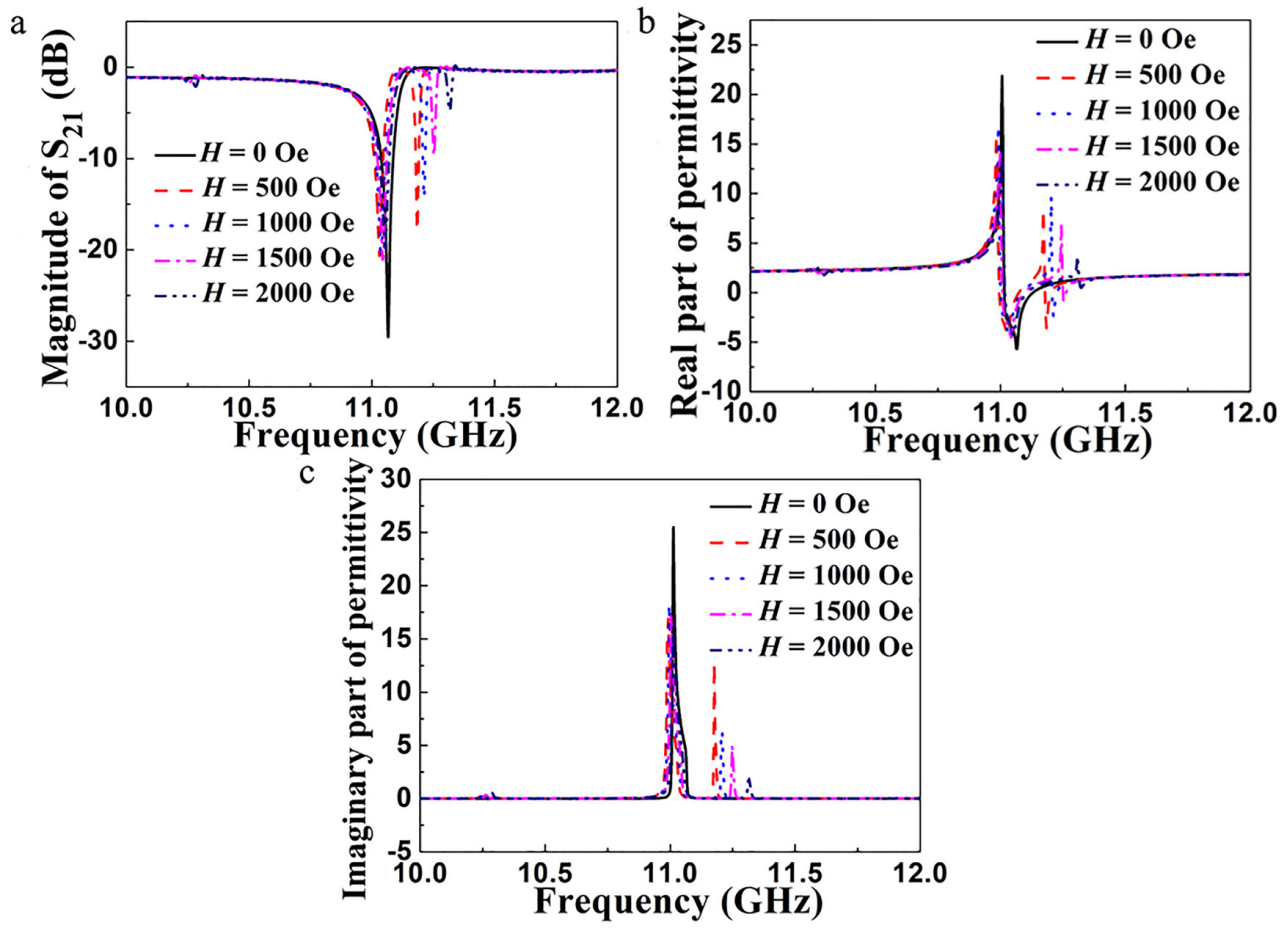
(a) Simulated transmission spectra for the unit cell of the metamaterial under a series of applied magnetic fields *H*. (b) Real parts and (c) imaginary parts of the effective permittivities retrieved from the simulated scattering parameters under a series of applied magnetic fields *H*.

Fig 3a shows the simulated transmission spectra for the unit cell of the metamaterial under a series of applied magnetic fields *H*. When *H* = 0, it can be seen that only one transmission dip appears at 11.07 GHz. Based on the analysis of magnetic field distributions as shown in Fig 2, the resonance mode corresponds to the electric Mie-resonance. When *H* = 500 Oe, one can see that two transmission dips appear at 11.03 GHz and 11.18 GHz, respectively. As *H* increases from 500 Oe to 2000 Oe, two transmission dips move to higher frequency region and the second transmission dip moves faster than the first one, which exhibits a better magnetically tunable behavior. The effective permittivity of the unit cell under the same series of applied magnetic fields *H* was extracted from the simulated scattering parameters [27–29]. Fig 3b shows the dependence of the calculated real part of effective permittivity on frequency. When *H* = 0, there is one remarkable dispersion in the range of 10–12 GHz. When *H* > 0, instead of one dispersion, two frequency dispersions appear in the range of 10–12 GHz, which also exhibits electric resonance characteristic. In addition, at a certain frequency region, the value of the effective permittivity is negative, which makes this metamaterial suitable for applications in negative refractive index materials. Fig 3c shows the dependence of the calculated imaginary part of effective permittivity on frequency. The imaginary part of the effective permittivity shows a similar behavior. From the above analysis, the effective permittivity of the metamaterial can be tuned by applied magnetic field.

**Fig 4:**
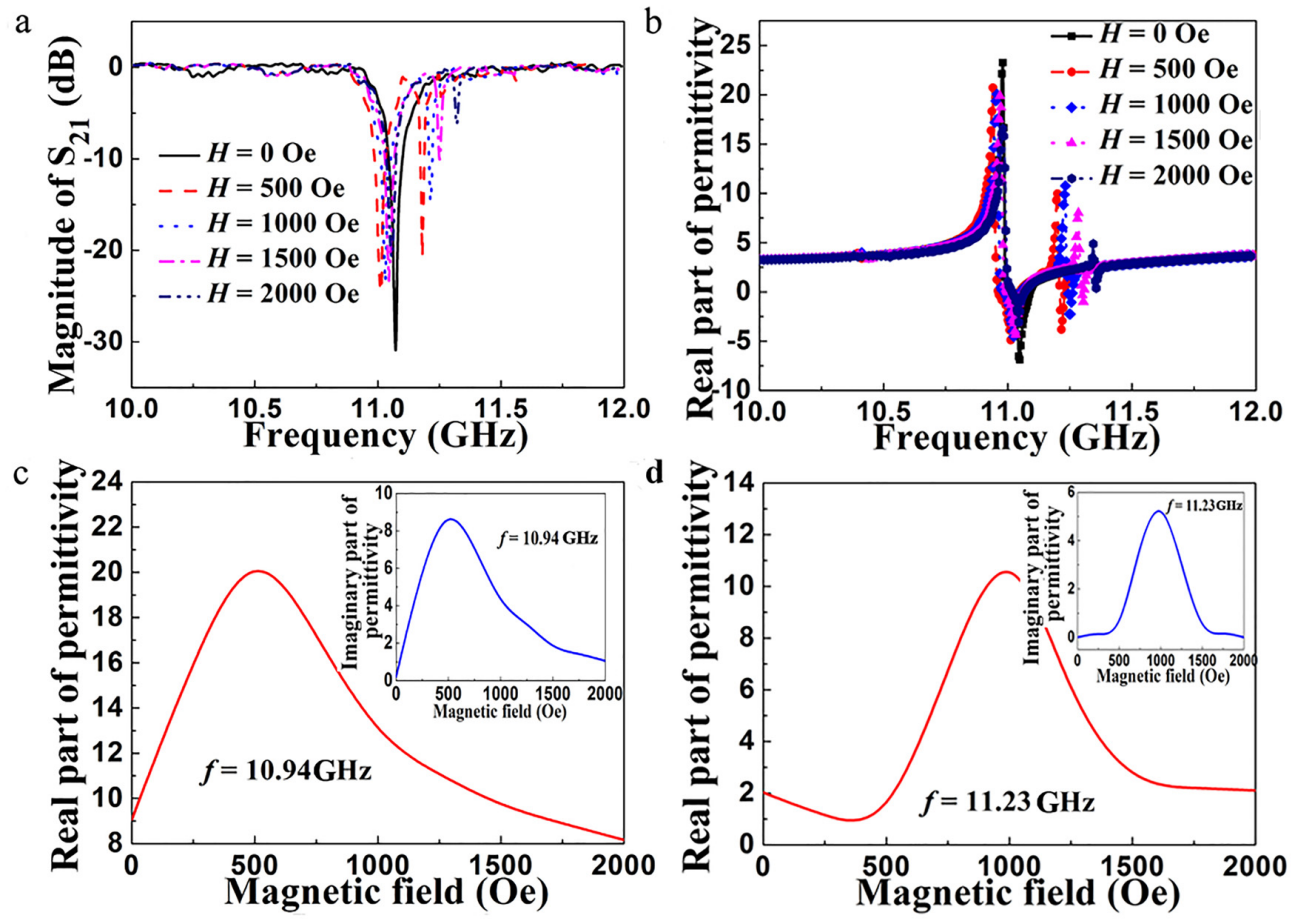
(a) Experimental transmission spectra for the metamaterial under a series of applied magnetic fields *H*. (b) Real parts of the effective permittivities retrieved from the experimental scattering parameters under a series of applied magnetic fields *H*. Magnetic field dependence of the real part of the effective permittivity of the metamaterial at (c) 10.94 GHz and (d) 11.23 GHz. The inset shows the dependence of the imaginary part of permittivity on the applied magnetic field.

Fig 4a shows the experimental transmission spectra for the metamaterial under a series of applied magnetic fields *H*. When *H* = 0, a transmission dip is induced by electric Mie-resonance of dielectric cuboid occurs in the transmission spectrum. When *H* > 0, two transmission dips appear in the range of 10–12 GHz. The behavior of the resonance frequencies is the same as that shown in Fig 3a, which exhibits a better magnetically tunable behavior. The typical electric Mie-resonance is induced by the dielectric cuboid, which results in one transmission dip appeared in the spectra. By interacting with the electromagnetic wave, the ferromagnetic precession takes place in the ferrite cuboid when *H* > 0. Based on Eqs (1–5), the permeability of the ferrite will changes, and then the permittivity of the metamaterial will be affected by the ferromagnetic precession which can be tuned by applied magnetic field. As shown in Fig 2b, the electric Mie-resonance in the dielectric cuboid is influenced by the ferromagnetic precession. Hence, two transmission dips appear in the transmission spectra and the resonance frequencies increase as *H* increases. The real parts of the effective permittivities retrieved from the experimental scattering parameters under the same series of *H* are depicted in Fig 4b. One Lorentz-type dispersion appears in the range of 10–12 GHz when *H* = 0 Oe. When *H* > 0, the second frequency dispersion appears, and the resonance frequency increases as *H* increases, which exhibits a magnetically tunable property. In addition, two negative permittivity frequency regions appear in the range of 10–12 GHz, which can be used to prepare dualband negative refractive index metamaterial. Fig 4c shows the magnetic field dependence of the real part of the effective permittivity of the metamaterial at 10.94 GHz. The inset shows the dependence of the imaginary part of permittivity on the applied magnetic field. The real part of the effective permittivity increases until it reaches a maximum (approximately 20.2) at *H* = 500 Oe and then decreases as *H* increases further. The imaginary part of the effective permittivity exhibits a similar behavior, and the value is relative small (8.6 at 10.94 GHz), which indicates the dielectric loss of this metamaterial is low. The magnetic field dependence of the effective permittivity of the metamaterial at 11.23 GHz is shown in Fig 4d. The behavior of the effective permittivity at 11.23 GHz is similar with that at 10.94 GHz. From the analysis presented above, it can be seen that the behavior of the experimental results is in good agreement with that of the simulated ones and the magnetically tunable dielectric property is much better than that in previous work (Ref. [22]).

## Conclusion

A ferrite-dielectric metamaterial composed of dielectric and ferrite cuboids has been prepared. When a certain magnetic field is applied, two resonance frequency dispersions appear and two peak values of the effective permittivity are obtained. By adjusting the applied magnetic field, the effective permittivity of the metamaterial can be tuned, which shows a magnetically tunable dielectric property. This work provides a new way to fabricate the tunable dielectric materials and negative refractive index metamaterials, which has greater potential for microwave devices.
